# Fostering Creativity in Intercultural and Interdisciplinary Teams: The VICTORY Model

**DOI:** 10.3389/fpsyg.2019.02020

**Published:** 2019-09-04

**Authors:** Min Tang

**Affiliations:** Institute for Creativity and Innovation, University of Applied Management, Ismaning, Germany

**Keywords:** interdisciplinary, intercultural, diversity management, team creativity, teaming

## Abstract

Teams are pervasive in the history of mankind. Particularly in our fast-growing modern society, teams composed of members from different cultures and disciplines are quite often used at the workplace. Though widely used, the effectiveness of teams is inconsistent. Meta-analyses report a double-edged effect of diversity on creativity and innovation, suggesting that diversity needs to be tactfully managed if we want to leverage the creative potential of teams. The current paper strives to meet this challenge and makes recommendations on how to foster creativity in intercultural and interdisciplinary teams. It discusses the concepts of teams vs. groups and creativity vs. innovation. Drawing upon sociocultural theories of creativity and innovation, particularly literature reviews and meta-analyses, this paper attempts to identify non-cognitive, cognitive and environmental enablers of team creativity. The VICTORY model offers a summary of these enablers, as it focuses on team (T) and synthesizes both non-cognitive (Vision, Openness, Risk-taking, Yes-I-Can Mindset) and cognitive (Ideation, Combination) antecedents of team creativity. Yet it is only through the combination and integration of environmental factors (including communication, collaboration, and support, among others) that the effect of these antecedents can be fully realized.

Teams have existed throughout histroy. From the primitive hunting period thousands of years ago to the highly connected modern global society of today, teams have been widely used to meet the various needs and challenges of human beings. It is because of the collective efforts of teams and groups that human beings are able to form cultures, create arts, and maintain development. As society advances further into the new millennium, the idea of teams will continue to evolve due to the fast development of technology and the increasing needs of integrating experiences across borders (Robbins and Finley, 2000).

Teams composed of members from different cultures and disciplines present potentials for creativity and innovation due to the possibility of integrating diverse perspectives, knowledge, skills, and abilities (Jackson, 1992; Williams and O’Reilly, 1998; West, 2002), and this diversity can stimulate creative thinking and prevent groups from moving prematurely to consensus on complex issues (van Knippenberg et al., 2004). However, diversity also poses possible risks, such as relational conflicts (De Dreu and Weingart, 2003), negative emotionality (Jehn, 1997; Jehn et al., 1999), stress (Donnellon, 1996; Jehn, 1997; Keller, 2001), or the possibility of “groupthink” (Janis, 1972), which hampers group cohesiveness and paralyzes team performance. Therefore, the double-edged effect of diversity on creativity and innovation needs to be carefully considered when using teams composed of members of diverse backgrounds to accomplish creative tasks.

The current paper focuses on how to build and manage interdisciplinary and intercultural teams to achieve creative goals. The first section summarizes and clarifies the definitions and distinctions between teams vs. groups and creativity vs. innovation. The second part reviews the literature of diversity and creativity/innovation, stressing the tactful trade-off of the double-edged effect of diversity on creativity/innovation in making the best of diversity for creativity/innovation in teams composed of members of diverse cultural and disciplinary backgrounds. The third section reviews and summarizes literature about team creativity and relates them to the interdisciplinary and intercultural approach that our university has been applying over the past decade in its research and training practice with the goal of fostering creativity in cross-disciplinary and multicultural teams. Finally, the strategies and methods are synthesized into a VICTORY Model, which draws on the sociocultural theory of creativity and covers both motivational and cognitive antecedents of team creativity.

## Teams vs. Groups

Teams are special types of groups where people work with commitment, shared responsibilities and complementary skills to achieve shared outcomes and common goals (Hackman, 1990; Cohen and Bailey, 1997). By contrast, groups are simply composed of two or more individuals who are influencing each other through interactions (Paulus, 1989; Forsythe, 1999). Though some scholars tend to conceptualize teams and groups interchangeably (e.g., Paulus and Van der Zee, 2004), the differentiation of these two concepts is preferred within the context of this paper for several reasons. First, flexibility and creativity in teams require strong commitment to the shared vision, purpose, and goals (Colenso, 1997), along with high degree of autonomy (Amabile, 1983), which are all defining characteristics of teams. Second, in creative teams people from different cultures and disciplines bring together substantial diversity. This requires more purposeful efforts to enable the functionality of diversity, which is essential for creativity (Hülsheger et al., 2009). Third, due to its premise of collective commitment, shared responsibilities and synergies of skills, teams seem to provide more appropriate basis for effective management of diversity to achieve innovative goals. Finally, the use of teams over groups is also consistent with the tradition and preference within the field of organizational psychology (Kozlowski and Ilgen, 2006).

It is worth mentioning that team creativity “encompasses both the *processes* of developing novel and useful ideas, as well as the new and appropriate *outcomes* that can be leveraged toward innovation” (Gilson et al., 2015, p. 178). However, researchers seem to forget that teams need to be built at the first place and they need to be motivationally, emotionally and cognitively prepared before intensive creative processes can take place and creative outcomes can be achieved. The VICTORY Model is developed to fill this gap to identify basic *non-cognitive*, *cognitive*, and *environmental* antecedents that a team would need for fruitful *creative processes*. In other words, it is a teaming (see Edmondson, 2012a,b) model which focuses on bringing members of diverse backgrounds together and preparing them for team creativity and innovation.

## Creativity vs. Innovation

Contemporary literature tends to define creativity as the production of new and useful ideas (e.g., Amabile, 1983, 1996; Mayer, 1999; Sternberg and Lubart, 1999; Zhou and Shalley, 2003) and innovation as comprising both the production of creative ideas and the implementation of the ideas (West and Farr, 1990; Amabile, 1996; Shalley and Zhou, 2008). Creativity is often regarded as the first step of innovation (Mumford and Gustafson, 1988; Amabile, 1996; West, 2002; Klijn and Tomic, 2010). As the creative process is cyclical and recursive (Paulus, 2002), and messy and reiterative (King, 1992), creativity actually occurs over the whole innovation implementation process. Acknowledging creativity and innovation as being integral parts of essentially the same process, Anderson et al. (2014) proposed an integrative definition of the two concepts:

“Creativity and innovation at work are the process, outcomes, and products of attempts to develop and introduce new and improved ways of doing things. The creativity stage of this process refers to idea generation, and innovation refers to the subsequent stage of implementing ideas toward better procedures, practices, or products.” (p. 1298).

The fact that people of different domains prefer using creativity over innovation or vice versa reflects the weight of their own subjective value of idea generation or the implementation of ideas (Werner et al., 2011). The current paper emphasizes the preparation of idea generation in diverse teams, which is closer to creativity. In the following part, theories of interdisciplinary and intercultural approach to creativity, sociocultural psychology of creativity, and diversity management will be reviewed before a model of team creativity will be introduced.

## Interdisciplinary and Intercultural Approach to Creativity

The interdisciplinary approach refers to the application of methods, knowledge, and modes of thinking from different *disciplines* to common questions or tasks, so that each discipline gains more than it would by working alone (Lindauer, 1998). Interdisciplinarity synthesizes diverse disciplines through intensive interaction and collaboration where the final result would be 1 + 1 > 2 (Klein, 1990, 2010). In business, interdisciplinary teams are frequently used to achieve innovative goals. For example, Ancona and Caldwell (1992) studied 45 new-product teams in five high-tech companies and found that the functional diversity and the frequent communication of the team members outside the work group’s boundaries led to higher levels of innovation. Another study of 100 health care teams found that greater numbers of professional diversities in the team led to higher levels of innovation (Borrill et al., 2000). Despite these findings, it is also important to caution that functional diversity can also result in communication barriers and misunderstanding (Eveland, 1987; Dougherty, 1992), and in addition threaten team’s safety or reduces team members’ clarity about the commitment to group objectives (West, 2002), which are detrimental to creativity and innovation. Therefore, effective management of diversity is essential to the implementation of creativity and innovation in diverse groups (Mumford and Gustafson, 1988; Tjosvold, 1998; Tang and Werner, 2017a).

In a similar vein, an intercultural approach can be defined as the application of methods, knowledge, and modes of thinking of different *cultures* to common questions or tasks, so that each culture gains more than it would by working alone. Creativity and innovation do not exist in a vacuum. Culture is the place where creativity and innovation are perceived, expressed, and evaluated (Ludwig, 1992; Tang and Werner, 2017a). Originality and usefulness have been widely accepted as the two defining determinants of creativity (for reviews, see Mayer, 1999; Lubart, 2010), but the evaluation of novelty/originality and usefulness/appropriateness are context-dependent and can differ from culture to culture (Rudowicz, 2003; Paletz and Peng, 2008; Lubart, 2010). The conceptions of creativity of the Eastern and Western culture are different. For example, sense of humor, aesthetic and art appreciation, which are consistently reported in North American implicit theories, are absent in the Chinese concepts of creativity (Rudowicz and Hui, 1997; Chan and Chan, 1999; Rudowicz and Yue, 2000). The creative modes also differ between cultures meaning that Eastern cultures more often follow the “S route” (S stands for spontaneous), which emphasizes adaptiveness, processes, intuitiveness, and metamorphism, whereas Western cultures prefer the “D route” (D stands for divergent), which emphasizes disruptiveness, results, rationality, and literalism (Elliot and Nakata, 2013). In addition, culture also has an influence on how people express or evaluate creativity (Niu and Sternberg, 2001; Yi et al., 2013; Tang et al., 2015) and whether they think creativity is fixed or trainable (Karwowski and Tang, 2016; Tang et al., 2016). The richness of culture and differences between cultures needs to be recognized and addressed in creativity training where participants from different cultures have an equal chance to share their perspectives and experiences on creativity and innovation.

## Sociocultural Psychology of Creativity and Diversity Management

Diversity is conceptualized as differences between individuals on any attribute that may lead to any possible dimension of differentiation (Jackson, 1992; Williams and O’Reilly, 1998; van Knippenberg and Schippers, 2007). Distinction is often drawn between two major diversity typologies: demographic and functional diversity. Demographic diversity refers to the readily observable differences related to gender, ethnicity, and age, whereas functional diversity is defined as the differences resulting from educational backgrounds or disciplines (see for a review, van Knippenberg and Schippers, 2007). In addition, attention has also been called to differences that do not fall into either of the two categories, such as differences in personality, attitudes, values (Harrison et al., 1998; Jehn et al., 1999), cognitive styles, and team membership change (Reiter-Palmon et al., 2012).

Although teams are popular within organizations and used extensively, results of meta-analyses on the effectiveness of diversity on creativity are inconsistent. Andrews’s (1979) meta-analyzed 1,222 research teams and found that diversity accounted for 10% of the variance in scientific recognition, R&D effectiveness, and number of publications, which are indicators of team creativity. However a more recent meta-analysis of over 30 years of team-level studies including 104 independent samples, found only a weak correlation between job-relevant diversity and team innovation (average *r* = 0.16), and also an insignificant negative correlation between background diversity and team innovation (average *r* = −0.13) (Hülsheger et al., 2009). Given these findings, diversity alone does not necessarily guarantee creativity and innovation. The double-edged effect of diversity on creativity and innovation needs to be tactfully managed in order to leverage the positive potential of teams.

The sociocultural psychology of individual creativity maintains that creativity involves an interaction of multiple factors both inside and outside the person, and that these *individual* and the *environmental* components must converge for creativity to occur (see a review, Tang, 2017). Since teams are composed of individuals, this approach can also apply to team creativity. Kerr and Tindale (2004) maintained that it is possible for team performance and decision-making processes to obtain gains or losses, both of which can be explained by situational and procedural factors that have an effect on motivation and resource coordination. A meta-analysis of over 100 independent teams (Hülsheger et al., 2009) identified 15 antecedents of team-level creativity including 9 team process variables, which are sociocultural variables in nature. Among them the most powerful environmental antecedents of innovative behavior of working teams are external communication (*ρ* = 0.475), support for innovation (*ρ* = 0.470), and internal communication (*p* = 0.358). Another literature review summarizes the antecedents of team creativity into three major categories: team composition (demographic diversity, functional diversity, cognitive style and personality, and team member change), social processes (team collaboration, communication, trust and psychological safety, backup and support, team conflict, cohesion, team efficacy), and cognitive processes (idea generation and brainstorming, creative problem solving, shared mental models, and team reflexivity) (Reiter-Palmon et al., 2012).

## The Victory Model

Existing studies suggest a double-edged effect of diversity on creativity and innovation. The advantages and disadvantages of using teams composed of members of diverse backgrounds need to be tactfully managed and balanced if we want to achieve high performances from teams. One crucial way to get positive effects out of diverse teams is to build and prepare teams systematically – not only cognitively but also motivationally, emotionally and environmentally. As mentioned above, the VICTORY Model is a “teaming model” which strives to bring members of diverse backgrounds together and prepare them for team creativity and innovation.

**Figure 1:**
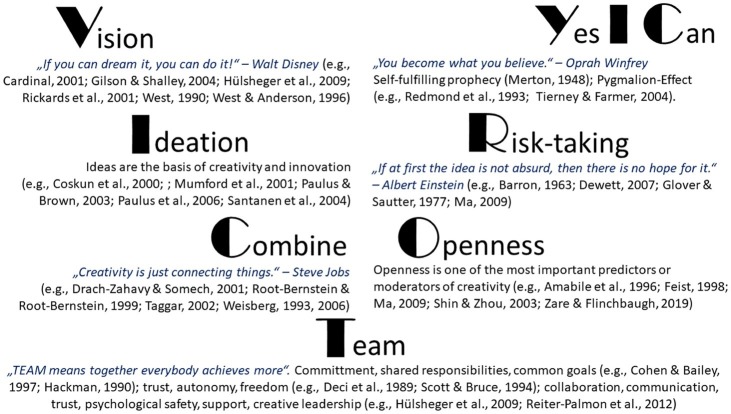
The VICTORY model for team creativity.

This model is developed by following two principles: the *parsimonious* principle and the *operational* principle. The *parsimonious* principle means that only a small number of basic factors, instead of all of them, will be selected. This principle helps readers, particularly practitioners, get focused instead of getting lost in the sea of components. To maximize the objectivity, this model draws upon classic theories of team creativity and the results of systematic reviews or meta-analyses. It is worth noting that such an approach is prone to publication bias (Rothstein et al., 2005), which will be discussed in detail in the limitation part. The *operational* principle means that the identified factors should be organized in an operational way so that readers, especially practitioners, can easily understand, remember, and implement the model. To make the model even more operational, concrete implementation examples from creativity research and training practice are provided. The VICTORY Model is summarized and presented in Figure 1.

The current paper serves two objectives: (1) to present a parsimonious and operational framework for achieving creativity in teams and (2) to discuss specific training solutions related to the model. In the following section, the meaning of each letter in the model will be explained using literature review/meta-analyses and the practical examples of creativity training.

## Teams – The Basis of the Victory Model

The VICTORY Model explicitly focuses on the team instead of on the individual level of creativity. By focusing on team creativity, we become fully aware of the potentials and challenges of translating the diversity of team members into creativity and innovation. We are aware that the relationship between diversity and creativity cannot be taken for granted, as meta-analyses have found only a weak correlation between job-relevant diversity and an insignificant negative correlation for background diversity and team innovation (Hülsheger et al., 2009). We know that simply putting people of different functional and cultural backgrounds together would not bring about creative and innovative outcomes. Instead, we as creativity trainers have the inescapable responsibility to “team” and facilitate individuals of diverse backgrounds to help them generate new ideas, find answers, and solve problems (Edmondson, 2012a). To achieve this goal, it is important to emphasize purpose, build psychological safety, embrace failure, and putting conflict to work (Edmondson, 2012b).

In our creativity training (e.g., Tang and Werner, 2017a,b), we taught the participants the differences between a loose group and a functional team. That is, “Team members share goals and are mutually held accountable for meeting them, they are interdependent in their accomplishment, and they affect the results through their interactions with one another. Since the team is collectively held accountable for the results, the work of interacting with one another is included among the responsibilities of each member” (Mohrman et al., 1995, pp. 39, 40). We integrate various tasks requiring different types of team collaboration. Some tasks require the participants to stay within their disciplines using domain knowledge to identify problems and challenges. Other tasks require the search for solutions through joint efforts across disciplines. Sometimes participants form national groups to explore problems from the cultural perspective. Sometimes they open the discussion with participants from other countries. These different types of tasks provide team members with the opportunities to work within or across disciplines and countries. We also adopted an approach called “Total Involvement Management” (for more details, see Tang and Werner, 2017a) to get every participant involved in the organization and management of the learning event. The rationale behind this is that people are more motivated and creative if they are given more trust, autonomy, and freedom in organizing themselves and accomplishing tasks (Deci et al., 1989; Scott and Bruce, 1994). This approach also involves the participant in making decisions. Participation in decision making has a positive effect on integration and commitment of individual team members (Locke, 1991; Heller et al., 1998). High level of integration and commitment are components of high intrinsic motivation, which has been proven to be fundamental to creativity, innovation, and invention (Amabile, 1996; Tang, 2010). Through this innovative measure, team members are motivated to cooperate pro-actively within and across cultures, disciplines, and work jointly to strive for innovative goals.

## Vision, Openness, Risk-Taking, and Yes-I-Can Mindset – The Non-Cognitive Antecedents

### Vision

In organizational psychology, vision is defined as “an idea of a valued outcome which represents a higher order goal and motivating force at work” (West, 1990, p. 310). The ability to visualize and communicate a bold future state for an organization has always been a vital component of successful leadership. Bill Gates predicted a future with computers on every desk and in every home at a time when computers were only used in big labs. Steve Jobs imagined the iPod and iPhone long before the world was addicted to them. History shows us abundant examples which help to prove the famous quote: “If you can dream it, you can do it!” In teams, the key function of a vision is the “clarity of and commitment to objectives” (West and Anderson, 1996, p. 682). A clear and inspiring vision should be developed and communicated to the team members to rally the people together, channel their efforts, and motivate them to carrying on with their creative endeavors (Gordon, 2017).

Vision has clear social connotations, as commitment is only possible if objectives and goals can be clearly articulated and communicated at the first place. Indeed, numerous studies have confirmed the decisive role of a shared vision to team creativity and innovation (e.g., West, 1990; West and Anderson, 1996; Cardinal, 2001; Rickards et al., 2001; Gilson and Shalley, 2004). Vision subsumes the concept of task orientation, which is “a shared concern with excellence of quality of task performance in relation to shared vision or outcomes” (West, 1990, p. 313). The commitment to team objectives and organizational goals and the sense of shared purpose and responsibility are also important precedents to team innovation (West, 1990; West and Anderson, 1996; Cardinal, 2001; Rickards et al., 2001; Gilson and Shalley, 2004). Hülsheger et al.’s (2009) meta-analysis of 104 independent studies involving 50,096 participants found that a shared vision (*ρ* = 0.493) was the strongest predictor of team innovation (surpassing other predictors such as external communication, support for innovation, and task orientation, among others).

In our practice of creativity training, we always imbed the creation of a team vision into the process of team-building. Participants from different disciplines and countries are mixed into groups. Then they are given a variety of challenging tasks which they need to apply creativity to solve. On the basis of the process and outcomes of the collective creative problem solving, team members will develop understanding of the characteristics of their creative DNA. Based on this, each team will develop a poster which contains a name, a logo and a slogan – all need to be creative – of the team. These posters serve as a simple, compelling and visualized vision of the team, which will guide the team in the subsequent joint efforts for creative tasks.

### Openness

Openness to experience is defined as a broad constellation of traits which contains *cognitive* (e.g., being imaginative, knowledgeable), *affective* (e.g., broad interest, need for variety), and *behavioral* (e.g., seeking sensations, actively trying new things) indicators (McCrae and Costa, 1987, 1997). Decades of systematic investigation on the relationship between personalities traits (such as Big Five investigations) including several meta-analyses have revealed one consistent result: Openness is one of the most important predictors of creativity (Feist, 1998; Ma, 2009; Zare and Flinchbaugh, 2019) and creative self-beliefs (Karwowski and Lebuda, 2016). At the organizational level, innovations are more likely to occur if the work teams and organization are perceived as being open to changes and new ideas, and furthermore if support for new ideas and their implementation is provided by managers, supervisors, and coworkers (Amabile et al., 1996; Madjar et al., 2002; Shin and Zhou, 2003).

In our creativity training, we take all cognitive, affective, and behavioral dimensions of Openness (McCrae and Costa, 1987) into consideration and explicitly encourage the participants to open the 3Hs – the *head* (being cognitively open, imaginative, original, and artistic); the *heart* (being affectively open, maintaining high curiosity, broad interest, and preference for complexity); and the *hand* (being behaviorally open, seeking sensations, taking risks, and actively trying new things). Perspective taking has been found to be an important mechanism to unlock the potential of diversity for team creativity (Hoever et al., 2012). Exercises are given to train the participants to respect the diversity and differences and to take perspectives of other disciplines and cultures. A safe, tolerant and encouraging environment is created where team members can effectively communicate and collaborate with each other.

### Risk-Taking

The creative process is an uncertain and uncomfortable process which is prone to various forms of risks, which may be motivational, emotional, cognitive, or economic. To create something new, one needs to step out of the comfort zone, defy the crowds, deviate from the social norms, and get ready for failure. No wonder Barron (1963) maintained that risk is *inherent* in the desire to create. Studies have provided empirical evidence to the importance of risk-taking to creativity. For example, Glover and Sautter (1977) discovered that when the level of risk-taking in groups of students increased, the students’ performance on the Torrance Test of Creative Thinking also increased in flexibility and originality, decreased in elaboration, and there was no change in fluency of responses. Dewett (2007) found that employees’ willingness to take risks was significantly related to both subjectively (*r* = 0.26) and objectively assessed creativity (*r* = 0.16) and this pro-risk attitude also fully mediated the effect of intrinsic motivation on R&D personnel. Ma’s (2009) meta-analysis has also confirmed the importance of risk-taking for creativity with a mean effect size of 0.13 (SD = 0.64).

Individuals are more willing to take risks if they feel safe, trusted, and supported by people around them. In organizational settings, team members who have strong feelings of belongingness and feel attached to other team members are more likely to cooperate, interact with each other, and exchange ideas (Hülsheger et al., 2009). Therefore, it is important for team members to build trust and a nonthreatening interpersonal climate (West, 1990) and to set up a supportive and cooperative work atmosphere where team members socialize, help each other and collaborate in problem solving (Amabile et al., 1996; Tiwana and McLean, 2005).

In our creativity training, we purposefully design unusual and even absurd activities (such as decorating the seminar room like a kindergarten, moving and dancing with bare feet, and talking to each other in all possible ways except language, etc.). The purpose of these activities is to move the participants away from their comfort zone and encourage them to take some risks to do things differently. Inspiring quotes, such as Albert Einstein’s “If at first, the idea is not absurd, then there is no hope for it” are used to encourage the participants to search for crazy and absurd ideas. Meanwhile, team members are trained to respect, support, and encourage each other (Amabile and Kramer, 2011) and to give creativity-conducive feedback (Zhou, 2008) to each other, which will make it easier for teams to take risks.

### Yes-I-Can Mindset

Oprah Winfrey’s famous saying “You become what you believe” has inspired many people to build self-confidence and to live their dreams. This saying has its scientific foundation in psychology, where it is referred to as a self-fulfilling prophecy (Merton, 1948), the fact that strongly held positive or negative beliefs may strongly influence people’s behavior, so that what people believe becomes reality. In literature, the self-fulfilling prophecy is also labeled as the Pygmalion Effect. McNatt’s (2000) meta-analysis of 17 independent studies provided support to a robust influence of the Pygmalion Effect on various performance criteria such as exam scores, physical output, and performance appraisals.

In organizational settings, the Pygmalion Effect takes effect through the interplay of the supervisor expectations of employee creativity, supervisor creativity-supportive behavior, employee perception of creativity expectation, and the creative self-efficacy of employees (Tierney and Farmer, 2004). Studies have shown that the higher the supervisor innovation expectations, the higher the level of the employees’ innovative behavior (Scott and Bruce, 1994); the more support the supervisors provide to employees’ creative behavior, the higher the creative self-efficacy of the employees; the higher the creative self-efficacy, the more focused and effective individuals will be able to use their cognitive resources, which can lead to more creativity at work (Redmond et al., 1993; Tierney and Farmer, 2002). The intricate Pygmalion process of how supervisor’s expectations are translated into changes in subordinate creativity was examined by Tierney and Farmer (2004). They found that higher expectations of creativity from supervisors lead to more creativity-supportive behavior, which result in more positive views on creativity expectations by employees, which in turn lead to higher creative self-efficacy of employees, which subsequently increase the creativity level of employees.

In our creativity training, we share the empirical evidence of the trainability of creativity with the participants (e.g., Rose and Lin, 1984; Scott et al., 2004a,b) and explicitly articulate our expectation of creativity on them. We give lectures about the foundations of creativity and innovation and show the participants that everybody can be creative in certain area in his/her own way, as creativity exists at different levels (Kaufman and Beghetto, 2009) and is demonstrated in different domains (Carson et al., 2005; Kaufman, 2012). We also provide the participants with different creative thinking, design thinking and creative problem-solving tools (details see the later section about “Ideation”) and give them the opportunity to apply these tools to solve challenging problems. Through this sort of training, the creative self-efficacy of the teams are boosted and a Yes-I-Can mindset is developed, which strongly support and facilitate the creative efforts of the teams.

## Ideation and Creative Combination – The Cognitive Antecedents

### Ideation

Team creativity involves a variety of cognitive processes happening at both the individual and team level. One of the most essential parts of these processes that is central to problem-solving in organizations is ideational creativity (Paulus et al., 2006). Ideational creativity involves generating, evaluating, and selecting novel ideas. Among others, brainstorming has been widely used for generating ideas in teams. Studies have shown that facilitated brainstorming with guidelines, instructions, and primes increase the fluency of idea generation in teams (Coskun et al., 2000; Paulus and Brown, 2003; Santanen et al., 2004). One weakness of the traditional face-to-face brainstorming is the process and idea loss due to waiting time and evaluation appreciation. To remedy this, adapted brainstorming techniques such as brainwriting (Rohrbach, 1969; Preiser, 2006) and electronic brainstorming (e.g., the group decision support system – GDSS) (Nunamaker et al., 1987) have been developed. Only the ability to generate ideas is not enough for innovation. Teams also need to have the ability to evaluate and select ideas for further development and implementation. This evaluation process can be complicated and problematic if teams are facing too many alternatives (Mumford et al., 2001). To increase the effectiveness of team idea evaluation and decision-making, some advice can be gained by empirical studies, such as increasing the group interaction (Larey and Paulus, 1999), taking sufficient time to evaluate all alternatives and include second thoughts (Kerr and Tindale, 2004), as well as developing the “shared mental models” (shared knowledge or beliefs) (Mumford et al., 2001).

In our team creativity training, we applied the 6-3-5 brainwriting method (Rohrbach, 1969) followed by a summative brainstorming session guided by the Osborn (1957)’s rules. The brainwriting was used before traditional oral brainstorming in order to cope with the possible side effects of brainstorming caused by social anxiety (Camacho and Paulus, 1995), waiting time (Nijstad et al., 2002) and social loafing (Karau and Williams, 1993). In addition, a variety of cognitive tools, such as the 13 creative thinking tools of Root-Bernstein and Root Bernstein (1999), the creative solving models (see a review, Treffinger and Isaksen, 2005), and the design thinking methods (e.g., those of the IDEO company) are also introduced and teams are encouraged to creatively combine different tools and methods to facilitate their creative processes.

### Creative Combination

Creative process in teams is defined as a collective endeavor of teams to behaviorally, cognitively, and emotionally define problems, generate ideas, and attempt new ways of going about their work (Gilson and Shalley, 2004). Laypersons usually hold mysterious views about the creative process and assume that creativity occurs through sudden spontaneous cognitive leaps and unconscious illuminations. Though this view seems pervasive in society, empirical evidence supporting this is lacking. Instead, based on a series of laboratory and real-world case studies of prominent scientists, artists, and inventors, Weisberg (1993, 2006) discovered that there was no such “out of the blue” creation. Rather, creative thinking begins with a continuity with the past – that is, what we already know – and goes beyond the past through reasoning and onward into the accumulation of new pieces of information. An excellent example of this theory is the iPhone, which has brought about revolutionary changes to the mobile phone business, the Internet economy and our society as well. Yet from a technical perspective, the iPhone was nothing more than a creative combination of different already existing functions or technologies (such as touchscreens, interface functionality, and cameras, etc.). The aspect that makes the iPhone so special and innovative is the synthetic effect arising from the meaningful combination of the existing antecedents: the iPhone is not only a mobile phone but also a mobile, portable computer.

In our training, we organize the participants to analyze a series of cases of revolutionary inventions or innovative products similar to iPhones. Gradually, participants realize that the creative process is nothing mysterious or elusive; rather, it starts with collecting information about what already exists. Studies have shown that in order to generate novel, useful suggestions for outcomes and achieve team innovation, information should be shared, combined and constructively dialogued (Drach-Zahavy and Somech, 2001; Taggar, 2002). In our training, we set up a schedule and framework for the various groups to meet on a regular basis to communicate, exchange ideas and share information. Grouping occurred on various levels: groups composed of members of the same culture, but different disciplines; groups composed of members of the same disciplines, but different cultures; or groups composed of members of different cultures and disciplines, but serving the same function in the program (such as media managers). Regular meetings of different forms and the open and safe environment enabled the team members to share information and communicate ideas, which facilitated the creative combination of ideas.

## The Interactive Nature of the Model

To invite awareness of the equal importance of factors on different dimensions, the components of the VICTORY Model can be classified into three main categories: (1) non-cognitive (Vision, Openness, Risk-taking, Yes-I-Can Mindset); (2) cognitive (Ideation, Combination); and (3) environmental (Team and the environmental enablers of the non-cognitive and cognitive components). In a team context, it is important to emphasize that all of these factors can also have sociocultural connotations. For example, a vision is inherently motivational as it represents a strong goal orientation and a motivating force (see the definition of West, 1990, p. 310). In teams, a vision must be clearly articulated and communicated until it becomes the shared vision (Hülsheger et al., 2009). In this sense, a shared vision becomes more sociocultural than purely motivational.

It is also important to consider that all components of the VICTORY Model are interactive with each other and are subject to the influence of the environment. For example, strong creative self-efficacy (i.e., the “Yes-I-Can” mindset) does not just develop on its own. It must be grounded on accumulative successful experiences with creativity. To enable the successful creative experiences, both cognitive (e.g., skills, heuristics, methods, etc.) and non-cognitive (e.g., motivation, personality, emotion, etc.) must converge so that individuals and teams will not only have the “will” but also the “ability” to create. In the interaction of non-cognitive and cognitive factors, environment plays an important role. For example, both Openness and Risk-taking are typical personality traits, but the degree of how much such personality traits can be demonstrated depends on how much the culture of an organization or the climate of the team would encourage and enable such personalities. Studies have shown that social process factors such as collaboration, communication, trust, psychological safety, support and creative leadership, etc. are important environmental facilitators for team creativity (for a review, see Reiter-Palmon et al., 2012).

## Limitations and Future Research

The VICTORY Model has been developed by following both parsimonious and operational principles so that researchers and practitioners can easily grasp key aspects of team creativity. However, these practical characteristics of the model also pose limitations. Firstly, literature reviews and meta-analyses, which lay the basis for the selection of the factors of the VICTORY Model, are prone to publication bias (Rothstein et al., 2005). This means that significant and positive results are more likely to be published than non-significant and negative results; this bias is a major threat to the validity of meta-analyses, as it can result in the overestimation of effect sizes. Nevertheless, a recent study of 83 meta-analyses on publication bias published in Psychological Bulletin and 499 systematic reviews from an authoritative medicine database found that evidence for publication bias in the studied homogeneous subsets was weak in both psychology and medicine (van Aert et al., 2019). This result means that the possible publication bias of meta-analyses should not undermine the merit of the current model, as the influence of the bias is only weak. Another limitation is that the VICTORY model, in its current form, is only structural but not processual. This limitation is due to the fact that the components of the model are highly interrelated and susceptible to the influence of the environment. The highly interactive feature of the components of the model makes it very challenging to differentiate the stages as each stage involves multiple components. In spite of this, it is worth the effort to explore the possible combination and sequences of the different components. Last but not least, though some evidence has already existed about the effect of training programs adopting the VICTORY Model (Tang and Werner, 2017a,b), more studies including well-designed experimental studies using comparable control groups are still needed to further validate the model.

## Conclusion

Gordon (2007) in his best-seller “The Energy Bus” emphasizes, “I truly believe that no one ever creates success alone. Everyone needs a positive team with supportive people at their side” (p. ix). Paulus and Van der Zee (2004) stress, “if we have to use teams, we should train and support them appropriately” (p. 477). The encouraging news is that studies have shown a moderate effect of training on creativity: a grand mean effect size of 0.47 based on 46 studies (Rose and Lin, 1984); an average Glass’ Δ of 0.68 based on 70 studies (Scott et al., 2004a); an average Glass’ Δ of 0.78 based on 156 studies (Scott et al., 2004b); and a grand mean effect size of 0.77 based on 34 studies (Ma, 2006). In addition, studies have also shown that well-designed creativity training can not only increase creativity but also people’s self-belief about their own creative ability – the so-called creative self-efficacy (e.g., Locke et al., 1984; Gist, 1989; Mathisen and Bronnick, 2009; Byrge and Tang, 2015; Tang and Joos, 2017; Tang and Werner, 2017a,b). Our 10 years’ experience with creativity training echoes the fact of the trainability of creativity. Our research and training are able to show that teams can be trained to be creative if the training is carefully designed to address not only the cognitive but also the non-cognitive and environmental factors. Through a longitudinal study composed of three times of measurement, our study has also provided evidence to the instant and sustained effect of team creativity training (Tang and Werner, 2017b).

The VICTORY Model is a summary of the team creativity intervention, which capitalizes on the diversity of **T**eam members and provides them with an **O**pen environment which respects diversity and encourages **R**isk-taking. Through bold but constructive risk-taking activities, teams learn how to jump out of their comfort zone and accumulate successful creative experiences. These accumulative experiences help the teams to strengthen their creative self-efficacy and develop a “**Y**es-I-Can”-mindset. Following the motivational, emotional, and personality preparation, teams are provided with a series of cognitive tools to help them form shared **V**isions, master basic **I**deation methods, and creatively **C**ombine existing ideas, processes, procedures, and products into new ones. Based on empirical studies (particularly meta-analyses) and a decade of continuous training practice, the VICTORY Model has been proven to be a powerful tool for teaming for creative purposes. Edmondson (2012b) maintains that “teaming is not just something individuals and companies have to do now but something they should want to do now, because it’s an important driver of personal and organizational development” (p. 79). By sharing the theoretical and practical essence of this model, I hope to help culturally and disciplinarily diverse teams to more effectively management their diversity, thus making the best out of this diversity to achieve creative and innovative goals.

## Author Contributions

The author confirms being the sole contributor of this work and has approved it for publication.

### Conflict of Interest Statement

The author declares that the research was conducted in the absence of any commercial or financial relationships that could be construed as a potential conflict of interest.
